# Herpes Simplex Virus Type-2 Cervicovaginal Shedding Among Women Living With HIV-1 and Receiving Antiretroviral Therapy in Burkina Faso: An 8-Year Longitudinal Study

**DOI:** 10.1093/infdis/jiv495

**Published:** 2015-10-15

**Authors:** Andrea J. Low, Nicolas Nagot, Helen A. Weiss, Issouf Konate, Dramane Kania, Michel Segondy, Nicolas Meda, Philippe van de Perre, Philippe Mayaud

**Affiliations:** 1 London School of Hygiene and Tropical Medicine, United Kingdom; 2 UMR 1058 University of Montpellier; 3 Montpellier University Hospital, France; 4 Centre Muraz, Bobo-Dioulasso, Burkina Faso

**Keywords:** antiretrovirals, HIV-1, herpes simplex virus type-2, HSV-2, female, genital tract, genital ulcer disease, Burkina Faso

## Abstract

***Background.*** The impact of antiretroviral therapy (ART) on herpes simplex virus type-2 (HSV-2) replication is unclear. The aim of this study was to assess factors associated with cervicovaginal HSV-2 DNA shedding and genital ulcer disease (GUD) in a cohort of women living with human immunodeficiency virus type-1 (HIV-1) in Burkina Faso.

***Methods.*** Participants were screened for cervicovaginal HSV-2 DNA, GUD, cervicovaginal and systemic HIV-1 RNA, and reproductive tract infections every 3–6 months over 8 years. Associations with HSV-2 shedding and quantity were examined using random-effects logistic and linear regression, respectively.

***Results.*** Of the 236 women with data on HSV-2 shedding, 151 took ART during the study period. Cervicovaginal HSV-2 DNA was detected in 42% of women (99 of 236) in 8.2% of visits (151 of 1848). ART was associated with a reduction in the odds of HSV-2 shedding, which declined for each year of ART use (odds ratio [OR], 0.74; 95% confidence interval [CI], .59–.92). In the multivariable model, the impact of ART was primarily associated with suppression of systemic HIV-1 RNA (adjusted OR, 0.32; 95% CI, .15–.67). A reduction in the odds of GUD was also observed during ART, mainly in those with HIV-1 suppression (adjusted OR, 0.53; 95% CI, .25–1.11).

***Conclusions.*** ART is strongly associated with a decrease in cervicovaginal HSV-2 shedding, and the impact was sustained over several years.

Herpes simplex virus type 2 (HSV-2) infection is one of the most common sexually transmitted infections, with the highest burden in Africa [1]. HSV-2 coinfection is associated with increased plasma and genital human immunodeficiency virus type 1 (HIV-1) loads [2, 3] and increased quantities of genital tract inflammatory cells [4, 5]. People living with HIV tend to have more-frequent HSV-2 clinical manifestations, with recurrent and persistent genital ulcerative disease (GUD) attributed to impaired immune responses [6, 7]. HIV coinfection has also been shown to increase genital shedding of HSV-2 [8] and the likelihood of transmission [9].

Antiretroviral therapy (ART) should reduce clinical and asymptomatic manifestations of HSV-2 infection, through immune restoration. The impact of antiretroviral therapy (ART) on GUD and HSV-2 shedding has been described in multiple contexts, with varying results depending on sampling frequency [10, 11]. Both GUD and HSV-2 genital shedding can increase during the first 1–3 months of ART, particularly among women with low CD4^+^ T-cell counts at ART initiation, likely owing to immune reconstitution [12, 13]. The impact of ART on HSV-2 shedding beyond 6 months has not been described.

In this article, we present data on the short-term and long-term effects of ART on symptomatic HSV-2 genital shedding (defined as the presence of GUD) and asymptomatic HSV-2 genital shedding in a cohort of high-risk women living with HIV-1 in Burkina Faso.

## METHODS

Participants were women living with HIV-1 and coinfected with HSV-2 who were enrolled in the Yerelon cohort in Bobo-Dioulasso, Burkina Faso [14–16]. Combined ART has been available since 2004 for women with World Health Organization clinical stage 3/4 HIV disease or a CD4^+^ T-cell count of ≤200 cells/µL (or ≤350 cells/µL, beginning in 2009) [17]. First-line treatment for most participants was based on nonnucleoside reverse transcriptase inhibitors. Participants were followed approximately every 3–6 months. A subset of women were enrolled in a randomized trial of valacyclovir to suppress HIV-1 genital shedding, with fortnightly visits over a 12-week period in 2004–2005 [18, 19]. All visits corresponding to regular cohort visits were included in this analysis, excluding those with valacyclovir use.

At each visit, a clinician performed a gynecological examination; recorded whether GUD was present, based on detection of vesicles or ulcers; and collected genital samples. Enriched cervicovaginal lavage (eCVL) was performed by infusing 2 mL of normal saline into the vagina for 60 seconds and collecting it into a cryotube. A swab was rotated 360 degrees in the cervical os and placed into the same cryotube [20]. Women with symptoms of reproductive tract infections were treated according to national syndromic management guidelines, which did not include acyclovir during the study period. Visits were deferred during menses.

The research protocol was approved by the institutional review boards at the London School of Hygiene and Tropical Medicine and Centre Muraz and the research ethics committee at the Burkina Faso Ministry of Health. All women provided written informed consent.

HSV-2 serology was assessed using the Kalon IgG2-ELISA kit (Kalon Biologicals). HIV-1 RNA in plasma and eCVL specimens was detected and quantified using real-time polymerase chain reaction (PCR) analysis (Generic HIV Viral Load; Biocentric) [21]. HSV-2 DNA was extracted from 200 µL of eCVL fluid by using the QIAamp DNA mini kit (Qiagen) and was eluted in 100 µL of buffer. HSV-2 DNA was amplified from 5 µL of eluate by TaqMan real-time PCR analysis, using the ABI Prism 7000 Sequence Detection Systems, and was quantified using the HSV-2 Quantitated External Control (Tebu-Bio) [22]. The lower limit of detection was 300 copies/mL (2.50 log_10_ copies/mL).

Cervical swabs were tested for *Neisseria gonorrhoeae* and *Chlamydia trachomatis*, using PCR (Amplicor CT/NG PCR assay; Roche); testing was restricted to swabs dating from 2007 onward, owing to the potential for DNA degradation [23]. Vaginal smears were examined using wet-mount microscopy. Bacterial vaginosis was diagnosed on the basis of the Nugent score assigned to heat-fixed vaginal smears. The presence of sperm was detected using qualitative PCR to detect the Y chromosome [24].

The frequency of GUD and HSV-2 shedding and the quantity of HSV-2 DNA were assessed after stratification by ART status and ART duration. HIV-1 RNA and HSV-2 DNA loads in plasma and eCVL specimens were transformed to log_10_ copies/mL. Viral suppression was defined as achieving an undetectable HIV-1 RNA load in plasma (defined as a plasma viral load of < 2.50 log_10_ copies/mL) within the first 12 months of ART, and immune reconstitution was defined as a CD4^+^ T-cell count increase of ≥100 cells/µL by 12 months after ART initiation [25]; data collected 18 months after ART initiation were evaluated if data collected at 12 months were missing. Logistic regression was used to estimate odds ratios (ORs) associated with (1) detectable shedding and (2) GUD, adjusting for within-woman correlation by using random-effects models. Multivariable logistic regression models were constructed using a hierarchical framework and included factors known to be associated with either GUD or detectable cervicovaginal HSV-2, namely age group [10, 26] and immune reconstitution [27, 28], or to be independently associated with GUD or HSV-2 DNA in univariable analysis, with a *P* value of <.10. Immune reconstitution and viral suppression were preferentially included in the final model owing to missing values for concurrent CD4^+^ T-cell counts and plasma viral load.

For the quantitative analyses, visits with undetectable HIV-1 or HSV-2 were assigned half the threshold value [18]. Random-effects linear regression was used to assess factors associated with the quantity of cervicovaginal HSV-2 DNA, restricted to visits with detectable HSV-2. A multiple linear regression model was constructed in the same fashion as the logistic model. Statistical analyses were performed using Stata, version 12.0 (StataCorp).

## RESULTS

Between 2003 and 2011, 317 women seropositive for HIV-1 and HSV-2 were enrolled, of whom 236 had data collected on cervicovaginal HSV-2, and 81 did not have any stored samples. The characteristics of women with shedding data are shown in Supplementary Table 1. The median age at cohort enrollment was 32 years (interquartile range [IQR], 18–48 years); 14% [33 of 236]) were receiving ART at their first visit during which HSV-2 DNA was measured, 54% (128 of 236) initiated ART during the study period, and 4% (10 of 236) did not have any HSV-2 DNA measured after starting ART. The median CD4^+^ T-cell count was 357 cells/µL (IQR, 196–564 cells/μL) at the first visit with HSV-2 DNA sampling and 177 cells/µL (IQR, 116–233 cells/μL) at ART initiation. The most common ART regimen was zidovudine/lamivudine/efavirenz (42%); 85% (130 of 151) achieved plasma HIV-1 suppression by 12 months of treatment, and 69% (104 of 151) achieved immune reconstitution.

Shedding was measured during 1896 cohort visits, with 1308 occurring during ART. There was a median of 11 visits (IQR, 1–16 visits) per woman during ART and 6 visits (IQR, 1–16 visits) per woman before ART initiation. The median follow-up time was 1.2 years (IQR, 0.2–1.7 years) before ART initiation and 6.2 years (IQR, 5.0–6.6 years) during ART; 48 visits at which women received valacyclovir were excluded from analyses.

**Figure 1.**
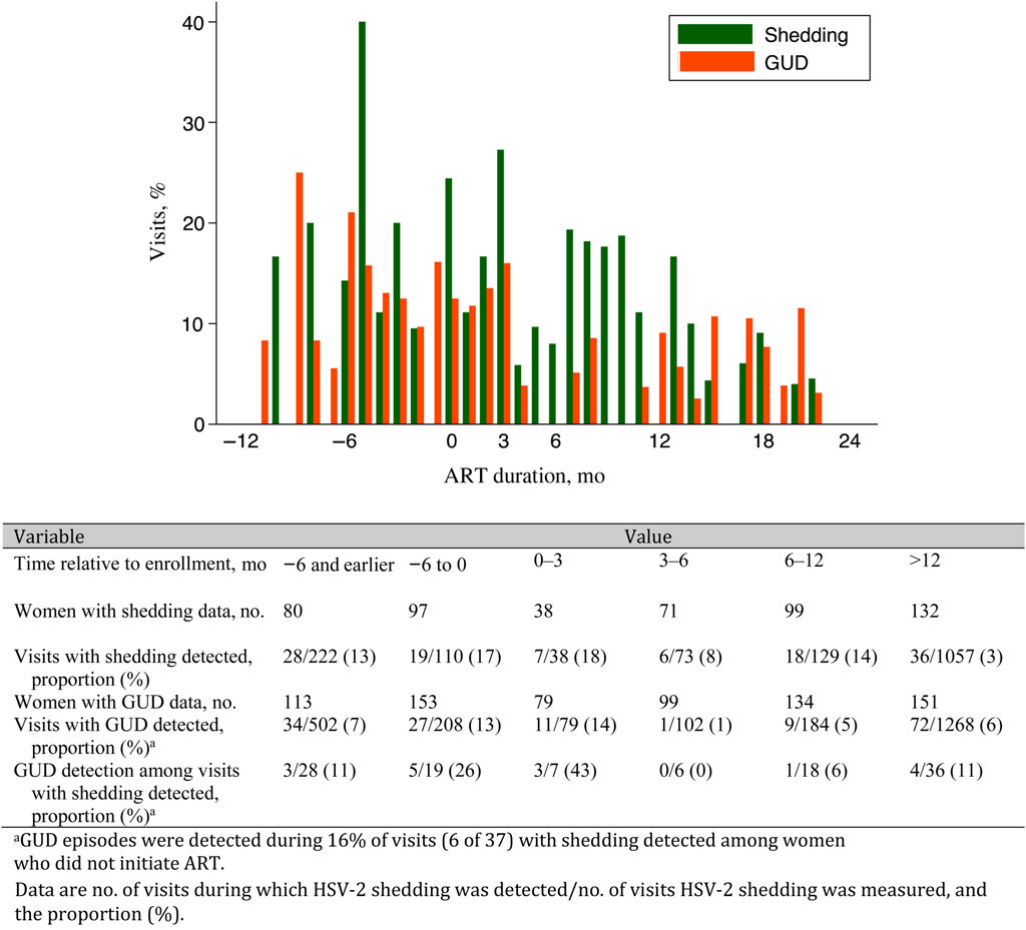
Frequency of genital herpes simplex virus type 2 (HSV-2) shedding and genital ulcer disease (GUD) among human immunodeficiency virus (HIV)–positive high-risk women before and after antiretroviral therapy (ART) initiation.

HSV-2 DNA was detected at least once in eCVL samples from 42% of women (99 of 236) at 8.2% of cohort visits (151 of 1848), with GUD detected concomitantly in 15% of shedding episodes (22 of 151). Of women with a measurement while not receiving ART, 33% (67 of 203) had detectable HSV-2 DNA at 15% of visits (84 of 551), and 32% (48 of 151) had detectable HSV-2 DNA at 5% of visits (67 of 1297; *P* < .001) after ART initiation (Table 1). The highest proportion of visits with shedding occurred during the last 6 months preceding ART (17% [19 of 110]; Figure 1) and the first 3 months of ART (18% [7 of 38]), and the proportion significantly dropped after 12 months of ART (3% [36 of 1057]; *P*_trend_ < .001). The proportion of visits during which GUD was detected also increased and decreased, during the same periods.

**Table 1.** Variables Associated With the Presence of Cervicovaginal Herpes Simplex Virus Type 2 (HSV-2) DNA and Genital Ulcer Disease (GUD) Among Women Living With Human Immunodeficiency Virus Type 1 (HIV-1) in Burkina Faso

Characteristic	HSV-2 DNA Presence									GUD Presence				
	Visits, Proportion (%) (n = 1848)^a^	OR (95% CI)^b^				Regression Coefficient^c^ (95% CI)				Visits, Proportion (%) (n = 2809)^a^	OR (95% CI)^b^			
		Unadjusted	*P* Value	Adjusted	*P* Value	Unadjusted	*P* Value	Adjusted	*P* Value		Unadjusted	*P* Value	Adjusted	*P* Value
Age, y^d^														
18–24	25/204 (12.3)	1.0		1.0		0		0		35/443 (7.9)	1.0		1.0	
25–34	78/874 (8.9)	0.58 (.28–1.18)		0.81 (.33–2.02)		0.09 (−.44 to .62)		0.38 (−.21 to .98)		70/1316 (5.3)	0.67 (.37–1.20)		0.53 (.23–1.22)	
≥35	48/767 (6.3)	0.39 (.19–.83)	.05	0.61 (.23–1.56)	.42	−0.16 (−.73 to .42)	.52	0.22 (−.42 to .86)	.44	69/1036 (6.7)	0.93 (.52–1.69)	.25	0.82 (.36–1.87)	.21
ART influence^e^														
ART status														
Naive	84/551 (15.3)	1.0		1.0		0		0		82/1176 (7.0)	1.0		1.0	
Initiated	67/1297 (5.2)	0.26 (.17–.39)	.001	0.34 (.20–.60)	.001	−0.51 (−.88 to –.14)	.007	−0.02 (−.57 to .53)	.94	93/1633 (5.7)	0.72 (.51–1.03)	.07	1.13 (.62–2.04)	.69
CD4^+^ T-cell count (per 100 cells/µL increase)	…	0.70 (.61–.81)	.001	…		0.04 (–.09 to .17)	.57	…		…	0.79 (.70–.89)	.001	0.86 (.74–1.01)	.06
Plasma HIV-1 RNA load (per log_10_ copies/mL) increase	…	1.70 (1.41–2.05)	.001	…		0.25 (.07–.42)	.005	0.13 (−.12 to .38)	.31	…	1.49 (1.23–1.79)	.001	…	
Immune reconstitution^f^														
No	51/502 (10.1)	1.0		1.0		0		…		60/772 (7.8)	1.0		…	
Yes	62/1111 (5.6)	0.54 (.31–.94)	.03	0.68 (.37–1.26)	.22	0.04 (−.39 to .47)	.22	…		93/1517 (6.1)	0.79 (.49–1.27)	.33	…	
Viral suppression^g^														
No	29/184 (15.8)	1.0		1.0		0		…		31/269 (11.5)	1.0		1.0	
Yes	78/1374 (5.7)	0.31 (.16–.62)	.001	0.32 (.15–.67)	.003	0.19 (−.31 to .70)	.45	…		117/1880 (6.2)	0.45 (.24–.83)	.01	0.53 (.25–1.11)	.09
Mucosal factor^e^														
Abnormal vaginal discharge														
No	89/1421 (6.3)	1.0		1.0		0		…		113/2154 (5.3)	1.0		1.0	
Yes	62/410 (15.1)	2.96 (2.00–4.38)	.001	2.86 (1.75–4.68)	.001	0.07 (−.31 to .46)	.71	…		62/655 (9.5)	1.99 (1.40–2.83)	.001	1.63 (.99–2.69)	.05
Bacterial vaginosis														
No	84/1198 (7.0)	1.0		…		0		…		92/1771 (5.2)	1.0		1.0	
Yes	59/580 (10.2)	1.38 (.94–2.04)	.10	…		−0.12 (–.52 to .28)	.57	…		74/927 (8.0)	1.60 (1.14–2.26)	.007	1.31 (.84–2.05)	.23
GUD														
No	129/1722 (7.5)	1.0		1.0		0		…		…	…		…	
Yes	22/108 (20.4)	3.22 (1.80–5.76)	.001	2.62 (1.28–5.38)	.008	−0.40 (–.94 to .13)	.14	…		…	…		…	
eCVL HIV-1 RNA														
No	82/1174 (7.0)	1.0		1.0		0		0		68/1399 (4.9)	1.0		1.0	
Yes	63/573 (11.0)	1.57 (1.06–2.33)	.02	0.82 (.48–1.38)	.45	0.50 (.12–.87)	.009	0.39 (−.11 to .89)	.13	58/677 (8.6)	1.73 (1.16–2.58)	.008	1.52 (.93–2.48)	.09

There was no association between the presence of HSV-2 DNA, the quantity of HSV-2 DNA detected, or the presence of GUD and the presence of a hemorrhagic sample, the presence of semen, ART regimen, or the presence of other reproductive tract infections.

Abbreviations: ART, antiretroviral therapy; CI, confidence interval; eCVL, enriched cervicovaginal lavage; OR, odds ratio.

^a^ Data denote no. of visits during which HSV-2 DNA or GUD was detected/total no. of visits. Denominators vary, owing to missing data.

^b^ ORs were calculated using random-effects logistic regression, and *P* values were determined by likelihood ratio tests.

^c^ Data are for visits during which HSV-2 DNA was detected. Regression coefficients were calculated using random-effects linear regression, and *P* values were determined by the Wald test.

^d^ Age was determined at cohort enrollment.

^e^ ART status and data on mucosal factors are concurrent to the cervicovaginal sample collection visit.

^f^ Immune reconstitution is defined as an increase in CD4^+^ T-cell count of ≥100 cells/µL 12–18 months after ART initiation. Data were available for 151 women receiving ART.

^g^ Viral suppression is defined as a viral load of <300 copies/mL within the first 12–18 months after ART initiation. Data were available for 144 women receiving ART.

Across all visits, concurrent CD4^+^ T-cell counts were associated with a 30% decrease in HSV-2 DNA detected per 100 cells/µL increase (OR, 0.70; 95% CI, .61–.81), whereas concurrent plasma HIV-1 loads were associated with a 70% increase per log_10_ copies/mL increase (OR, 1.70; 95% CI, 1.41–2.05). ART use was associated with a substantial reduction in HSV-2 shedding (OR, 0.26; 95% CI, .17–.39). There was a 25% decrease in the odds of shedding per year of ART after the first 12 months (OR, 0.74; 95% CI, .59–.92). In the multivariable model, HIV suppression was associated with a reduced odds of HSV-2 shedding (adjusted OR, 0.32; 95% CI, .15–.67; Table 1). Shedding episodes decreased with age in the univariable analysis, although this relationship was less pronounced after adjustment for ART (older women being more likely to be receiving ART).

During visits with HSV-2 shedding, the mean quantity of HSV-2 DNA was 4.47 log_10_ copies/mL (95% CI, 4.20–4.73; n = 84) in ART-naive participants and 3.96 log_10_ copies/mL (95% CI, 3.70–4.22; n = 67) after ART initiation (regression coefficient, −0.51; 95% CI, −.88 to −.14; *P* = .007; Table 1). Concurrent plasma HIV-1 loads were strongly associated with an increase in the amount of HSV-2 DNA shed (regression coefficient, 0.25 per log_10_ increase in PVL; 95% CI, .07–.42). In the multivariable model, there was weak evidence that concurrent HIV-1 shedding was predictive of an increased quantity of HSV-2 DNA (adjusted regression coefficient, 0.39; 95% CI, −.11 to .89).

There were 317 women with clinical data on GUD during the study period. GUD was present in 22% of women (65 of 292) with data prior to ART initiation and in 32% (92 of 192) with data after ART initiation; among visits, 7.0% (82 of 1176) before ART initiation and 5.7% (93 of 1633) after initiation revealed GUD (*P* = .17). There were concurrent vesicles at 43% of visits (23 of 124) with ulcers. HSV-2 DNA was detected at 20% of visits (22 of 108) with GUD present and 7.5% of visits (129 of 1722) without GUD present (*P* < .001). Overall, there was a decrease in frequency of GUD episodes with an increase in concurrent CD4^+^ T-cell count (OR, 0.79; 95% CI, .70–.89 per 100 cells/µL increase) and an increase with increasing plasma viral load (OR, 1.49; 95% CI, 1.23–1.79 per log_10_ copies/mL increase). There was an increase in the odds of GUD during the first 3 months of ART (OR, 2.00; 95% CI, .96–4.20) but an overall reduction in the odds of GUD during ART (OR, 0.72; 95% CI, .51–1.03). In the multivariable model, there was weak evidence that GUD was inversely associated with HIV-1 suppression (adjusted OR, 0.53; 95% CI, .25–1.11) and with increasing CD4^+^ T-cell count (adjusted OR, 0.86; 95% CI, .74–1.01).

## DISCUSSION

We describe the impact of ART on cervicovaginal HSV-2 and GUD presence over several years. The frequencies of HSV-2 shedding and GUD increased in the 6 months prior to ART initiation, were sustained at that level for the first 3 months of ART, and decreased thereafter. This differs from findings from a study in Uganda, where there was a rise in the frequencies of HSV-2 shedding and GUD during the first 3 months of treatment [13]. In our study, the most substantial decrease in shedding was seen after 12 months of ART, although we were limited by the small number of samples in the first 3 months. The effect of ART was associated with HIV-1 suppression and immune reconstitution, although the magnitude of the effect was larger for viral suppression. This further supports the synergistic interactions between HIV-1 and HSV-2 replication, where systemic HIV-1 replication might drive HSV-2 replication in the sacral ganglia, compounded by weak immune control [29–31]. This reduction was maintained over time and was independent of age; therefore, it is less likely to be due only to the natural history of HSV-2 [9]. Among women who shed, HSV-2 DNA quantities were correlated with quantities of genital HIV-1 RNA, providing additional proof of local direct viral interactions [32].

The effect of ART on GUD appears to be driven by systemic HIV-1 suppression, although there was a decrease in the frequency of GUD among women with higher CD4^+^ T-cell counts and a trend toward a reduction in the odds of GUD among women with immune reconstitution during ART. The slightly different dynamics for the effect of ART on GUD, compared with HSV-2 shedding, suggest that the clinical benefits might wane over time.

This is one of the first studies to demonstrate prolonged suppression of HSV-2 shedding during ART; other studies have shown no change in shedding during ART but reductions in GUD [33, 34]. The variations in results are likely due to smaller sample sizes and variable duration of follow-up, particularly if studies are limited to early periods after ART initiation.

There are limitations to this study. The frequency of sampling was every 3–6 months, and therefore clinical and asymptomatic episodes of HSV-2 activation might have been missed. GUD was assumed to be caused mainly by HSV-2 in this population, based on studies from the region [35, 36]. Our prior study in this population showed that 52% of GUD cases harbored lesional HSV-2 DNA [7]. Although we only detected HSV-2 DNA at 20% of visits with concurrent GUD, this is consistent with other studies that used more sensitive methods [10, 37].

In conclusion, ART has a significant influence on HSV-2 shedding and GUD episodes, primarily associated with HIV-1 suppression. Following ART initiation, HSV-2 shedding is rapidly suppressed, and the influence of ART is sustained over time.

## STUDY GROUP MEMBERS

Members of the Yérelon study group are as follows: Eloi Bahembera, Abdramane Berthé, Minata Coulibaly, Marie-Christine Defer, Ramata Diallo, Didier Djagbaré, Charlotte Huet, Issouf Konaté, Florent Ky-Dama, Gilles T. M'Boutiki, Nicolas Méda, Inès Millogo, Nicolas Nagot, Abdoulaye Ouédraogo, Djénéba Ouédraogo, Francois Rouet, Anselme Sanon, Haoua Sawadogo, Roselyne Vallo, and Laurence Vergne (deceased January 2007; Centre Muraz, Bobo-Dioulasso, Burkina Faso); Philippe Mayaud and Helen A. Weiss (London School of Hygiene and Tropical Medicine, United Kingdom); Nicolas Nagot, Pierre Becquart, Vincent Foulongne, Michel Segondy, and Philippe Van de Perre, (Université Montpellier 1 and CHU Montpellier, France); and Jean-Baptiste Andonaba and Adrien Sawadogo (University Hospital of Bobo-Dioulasso, Burkina Faso).

## Supplementary Data

Supplementary materials are available at http://jid.oxfordjournals.org. Consisting of data provided by the author to benefit the reader, the posted materials are not copyedited and are the sole responsibility of the author, so questions or comments should be addressed to the author.

Supplementary Data
