# Supplementary material for: Herpes Simplex Virus Type-2 Cervicovaginal Shedding Among Women Living With HIV-1 and Receiving Antiretroviral Therapy in Burkina Faso: An 8-Year Longitudinal Study
Source: J Infect Dis. 2015 Oct 15;213(5):731–7. doi: 10.1093/infdis/jiv495 (PMC4747618; doi:10.1093/infdis/jiv495)
Supplement: Supplementary Data [file supp_213_5_731__index.html]

Herpes simplex virus type-2 (HSV-2) cervico-vaginal shedding among women living with HIV-1 on antiretroviral therapy in Burkina Faso: an 8-year longitudinal study — Herpes Simplex Virus Type-2 Cervicovaginal Shedding Among Women Living With HIV-1 and Receiving Antiretroviral Therapy in Burkina Faso: An 8-Year Longitudinal Study — Herpes Simplex Virus Type-2 Cervicovaginal Shedding Among Women Living With HIV-1 and Receiving Antiretroviral Therapy in Burkina Faso: An 8-Year Longitudinal Study — Supplementary Data 

# Herpes Simplex Virus Type-2 Cervicovaginal Shedding Among Women Living With HIV-1 and Receiving Antiretroviral Therapy in Burkina Faso: An 8-Year Longitudinal Study

## Supplementary Data

Supplementary Data

- Supplementary Data - Doc file
