# Supplementary material for: Herpes Simplex Virus Type-2 Cervicovaginal Shedding Among Women Living With HIV-1 and Receiving Antiretroviral Therapy in Burkina Faso: An 8-Year Longitudinal Study
Source: J Infect Dis. 2015 Oct 15;213(5):731–7. doi: 10.1093/infdis/jiv495 (PMC4747618; doi:10.1093/infdis/jiv495)
Supplement: Supplementary Data [file supp_jiv495_jiv495supp.doc]

**Supplementary Table 1. Baseline characteristics of cohort participants with data on cervico-vaginal HSV-2 shedding**

| **Characteristic** | **Value^a^**  (N=236) |
| --- | --- |
| Age at enrolment – years (%)  Median (IQR)  18-24  25-34  ≥35 | 32 (18-48)  34 (15)  111 (47)  89 (38) |
| Full time sex worker at enrolment (%) | 31/235(13) |
| Median age at first sex – years (range) | 16 (10-23) |
| Median age at first client – years (range) | 19 (10-39) |
| Mean number of clients in the past week (±SD) | 6 (±14.5) |
| Report a history of ulcers or vesicles (%) | 82/236 (35) |
| Syphilis seropositive (%) | 4/199 (2) |
| Number receiving ART at 1^st^ visit with HSV-2 DNA measured (%) | 33 (14) |
| Pre-ART CD4 + count (%), cells/μl (N=137)  Median (IQR)  <200  200-499  >500  Median pre-ART CD4+ count (IQR) | 357 (196-564)  93 (68)  43 (31)  1 (1)  177 (116-233) |
| Mean plasma HIV-1 RNA pre-ART, log_10_ copies/ml (±SD) (N=134) | 4.88 (±0.94) |
| Detectable cervico-vaginal HIV-1 RNA at pre-ART visit (%)  Mean cervico-vaginal HIV-1 RNA^b^ -log_10_ copies/ml (±SD) | 101/131 (77)  3.90 (±0.78) |
| Detectable cervico-vaginal HSV-2 DNA at pre-ART visit (%)  Mean cervicovaginal HSV-2 DNA^c^ log_10_ copies/ml (±SD) | 19/128 (15)  4.29 (±1.05) |
| **First line ART regimen (%) (N=151)**  Efavirenz/Zidovudine/Lamivudine  Efavirenz/Stavudine/Lamivudine  Nevirapine/Zidovudine/Lamivudine  Nevirapine/Stavudine/Lamivudine  Protease inhibitor or Other | 63 (42)  22 (15)  17 (11)  46 (30)  3 (2) |

**NOTE**. The baseline visit is either at cohort enrolment (indicated) or at the first visit with HSV-2 DNA shedding data. The pre-ART visit is the last visit before initiation of ART. HSV-2, herpes simplex virus type 2; IQR, interquartile range; SD, standard deviation; ART, antiretroviral therapy.

^a^ Denominators vary due to missing data.

^b,c^ Among women with detectable HIV-1 RNA or HSV-2 DNA shedding respectively.
